# Clinical usefulness of conversion surgery for unresectable pancreatic cancer diagnosed on multidetector computed tomography imaging: Results from a multicenter observational cohort study by the Hokkaido Pancreatic Cancer Study Group (HOPS UR‐01)

**DOI:** 10.1002/ags3.12272

**Published:** 2019-07-09

**Authors:** Yasutoshi Kimura, Toru Nakamura, Tsuyoshi Hayashi, Masaki Kuwatani, Masayo Motoya, Makoto Yoshida, Masafumi Imamura, Minoru Nagayama, Hiroshi Yamaguchi, Keisuke Yamakita, Takuma Goto, Yusuke Sakuhara, Kuniyuki Takahashi, Hiroyuki Maguchi, Satoshi Hirano, Ichiro Takemasa

**Affiliations:** ^1^ Department of Surgery Surgical Oncology and Science Sapporo Medical University School of Medicine Sapporo Japan; ^2^ Hokkaido Pancreatic Cancer Study Group; HOPS Sapporo Japan; ^3^ Department of Gastroenterological Surgery II Hokkaido University Faculty of Medicine Sapporo Japan; ^4^ Center for Gastroenterology Teine‐Keijinkai Hospital Sapporo Japan; ^5^ Department of Gastroenterology and Hepatology Hokkaido University Graduate School of Medicine Sapporo Japan; ^6^ Department of Gastroenterology and Hepatology Sapporo Medical University School of Medicine Sapporo Japan; ^7^ Department of Medical Oncology Sapporo Medical University School of Medicine Sapporo Japan; ^8^ Division of Metabolism and Biosystemic Science Department of Medicine Asahikawa Medical University Sapporo Japan; ^9^ Division of Gastroenterology and Hematology/Oncology Department of Medicine Asahikawa Medical University Sapporo Japan; ^10^ Department of Radiology Tonan Hospital Sapporo Japan

**Keywords:** locally advanced, metastatic, multidisciplinary treatment, radical surgery, unresectable pancreatic cancer

## Abstract

**Background and Aim:**

Effective multidisciplinary approaches for unresectable pancreatic cancer (UR‐PC) that include modern chemotherapeutic regimens and subsequent conversion surgery (CS) are being developed. The aim of this study was to evaluate outcomes of patients clinically diagnosed with UR‐PC, focusing on the efficacy of CS.

**Methods:**

Patients ineligible for two multicenter phase II studies conducted by the Hokkaido Pancreatic Cancer Study Group (HOPS) were recruited. Sequential treatment regimens, conversion to radical surgery, and overall survival (OS) were analyzed by multidetector computed tomography (MDCT)‐based UR factors. Univariate and multivariate analyses were performed to identify predictors of OS.

**Results:**

Sixty‐six of 247 intended recruits for HOPS studies from October 2013 to April 2016 were included. Unresectability was due to locally advanced (LA) disease and metastasis (M) in 42 and 24 patients, respectively. Induction therapy began with chemotherapy (CT) and chemoradiotherapy (CRT) in 44 and 17 patients, respectively, of whom 23 received modern CT regimens. Radical surgery was completed in 12 (LA, 10; M, two) with a median treatment interval of 10.3 months (range, 2‐32). Eleven patients (91.6%) achieved pathological R0 resection. Median OS was significantly longer in patients who underwent CS than those who did not (44.1 vs 14.5 months, *P *< 0.0001). CS was an independent predictor of OS (hazard ratio, 0.078; 95% confident interval, 0.017‐0.348; *P *=* *0.001).

**Conclusion:**

Conversion surgery after a favorable response to sequential treatment might prolong survival in patients with UR‐PC. Precise diagnosis on MDCT followed by sequential multimodal anticancer treatment is essential.

## 1 INTRODUCTION

Pancreatic cancer (PC) is one of the most aggressive malignancies.^1^ In 2018, there were 44 330 estimated patients with PC‐related death in the USA and 34 990 patients in Japan, suggesting that PC is the fourth leading cause of cancer‐related death in both countries.^1^, ^2^

In the National Comprehensive Cancer Network (NCCN) guidelines,^3^ resectability is categorized as resectable (R), borderline resectable (BR), or unresectable (UR) based on multidetector computed tomography (MDCT) evaluation. Upfront surgery followed by postoperative adjuvant therapy was generally recommended for potentially resectable PC (R‐PC)^3^, ^4^ as well as neoadjuvant treatment followed by surgery for BR‐PC in order to achieve R0 resection.^3^ Despite marked improvements in diagnostic modalities, PC often presents as a systemic disease, which precludes early detection. More than 80% of patients are diagnosed with UR because of its high metastatic (M) potential.^5^ Recent advances in anticancer treatment for locally advanced (LA) UR, or M‐PC facilitate good disease control; such patients sometimes convert to surgical resection.^6^ This surgical strategy is called conversion surgery (CS).^7^ Several reports on CS in patients with UR‐PC have shown that it has a favorable effect on overall survival (OS).^6^, ^8^, ^9^ In recent meta‐analyses of reports from 2009 to 2015, the rate of conversion from UR‐LA‐PC to surgery was 26% and OS ranged from 18.7 to 24.2 months.^10^, ^11^ The entire cohort examined in these meta‐analyses comprised patients recruited into clinical trials conducted before 2013.

The Hokkaido Pancreatic Cancer Study Group (HOPS) conducted multicenter phase II studies to investigate the efficacy of neoadjuvant treatment for BR‐PC and R‐PC. To analyze the data from patients with UR‐PC whose diagnosis was based on central review of MDCT findings but were ineligible for these two HOPS studies, we conducted a multicenter study. Since those patients were managed at referral hospitals in Hokkaido prefecture thereafter, their survival data were recognized as real‐world patient outcomes.

## 2 METHODS

### 2.1 Study design

In this multicenter, retrospective study by HOPS, we assessed the outcomes of patients clinically diagnosed with UR‐PC and treated at tertiary referral hospitals around Hokkaido prefecture. The institutional review boards of Sapporo Medical University Hospital (282‐39, University Hospital Medical Information Network Clinical Trials Registry, UMIN000035454) and each participating hospital approved the study protocol.

### 2.2 Patients

Hokkaido Pancreatic Cancer Study Group conducted two multicenter phase II studies to investigate the efficacy of neoadjuvant treatment for R‐PC and BR‐PC (UMIN 000013031/000012293). They involved neoadjuvant chemotherapy (CT) consisting of two cycles of S‐1 (80 mg/m^2^, twice daily) or neoadjuvant chemoradiotherapy (CRT) with a total dose of 50.4 Gy in 28 fractions plus S‐1 (80 mg/m^2^, twice daily on radiation days) followed by subsequent gemcitabine CT for three cycles. These two studies recruited 247 patients with PC who were intended to participate from October 2013 to April 2016. Dynamic MDCT from the chest to the pelvis was performed to determine resectability based on the NCCN guidelines, version 2.2012^12^ through central review by HOPS. In brief, celiac abutment of pancreatic head cancer, arterial encasement of more than half of the circumference in other situations, and metastatic disease were categorized as UR. Regarding the common hepatic artery, a diagnosis of LA‐UR disease was made when safe, complete resection and reconstruction were very difficult because of tumor extension to the bifurcation of the hepatic artery.^8^ All MDCT interpretations were performed by two radiologist (Y.S. and D.A.) and verified by a surgeon (T.N.) and a gastroenterologist (M.K.). Suspicious liver metastasis on MDCT was confirmed with gadoxetic acid‐enhanced magnetic resonance imaging (EOB‐MRI), contrast‐enhanced ultrasonography, or both. Positron emission tomography (PET)‐CT was used for confirming other types of metastasis. Peritoneal metastasis was diagnosed when there was an intra‐abdominal nodule or mass separate from the pancreas located on the surface of the peritoneum, greater omentum, or intestine. Peritoneal metastasis was suspected if an area with the density of water was found in the abdominal cavity on MDCT, for example, in the pouch of Douglas, paracolic gutter, or around the liver or the surface of the spleen. Patients defined as having UR disease were ineligible for these two studies and based on HOPS central MDCT review were introduced into this retrospective study. In general, the diagnosis of PC was confirmed by endoscopic ultrasound‐guided fine‐needle aspiration or brush cytology during endoscopic retrograde cholangiopancreatography.

The attending physician suggested CS to patients with UR‐PC who met the following conditions: (a) treatment effect was evaluated as stable disease (SD), partial response (PR), or complete response (CR) based on Response Evaluation Criteria in Solid Tumors (RECIST) version 1.1^13^ after ≥ approximately 6 months of preoperative treatment; (b) R0 resection was deemed possible based on multimodal imaging; (c) resection of distant metastatic lesions when they showed no progression during preoperative treatment and could be resected completely. If all the metastases were treated with preoperative treatment, resection of the primary lesion alone was performed.^8^

### 2.3 Assessment

Clinical treatment effect was assessed using RECIST version 1.1.^13^ The histologic assessment of the extent of preoperative treatment response was evaluated using the Evans grading system.^14^ The Clavien‐Dindo classification was used to assess postoperative complications.^15^ Mortality was defined as death during the hospital stay when surgery was performed. Individual survival was defined as the duration between the date of treatment initiation and death or latest hospital visit. Median follow‐up was defined as the duration between the date of MDCT consultation and the latest hospital visit for censored patients.

Data collection was conducted at three times. The first survey was from July to October 2016. The second survey was from May to June 2017. The final survey was from March to April 2018. Survival data of patients was fixed on April 29, 2018. At that time, 51 events had occurred in 66 patients, including 32 patients with UR‐LA disease and 19 with UR‐M disease. Median follow‐up for censored patients was 35.6 months (range, 1.1‐47.9).

### 2.4 Outcome measures and statistical analysis

Variables included individual patient data, imaging findings, diagnostic information including cTNM stage according to the General Rules for the Study of Pancreatic Cancer (July 2016, seventh edition),^16^ cytology or histology results, date of MDCT consultation, type of biliary drainage, details on sequential treatment regimens, details about attempted radical surgery, pathological findings, and outcomes including disease recurrence or death.

Outcome measures included sequential treatment regimens, conversion to radical surgery, and OS. Outcome measures were analyzed by MDCT‐based UR factors including LA and M disease. Univariate and multivariate analyses were performed to determine predictors of OS.

Comparisons between two groups were performed using the Chi‐squared test, Mann‐Whitney *U* test, or Cox proportional hazards regression modeling for nonparametric data. Factors with *P *<* *0.2 on univariate analysis without potential confounding were included in multivariate logistic regression models to calculate adjusted odds ratios. OS was calculated using the Kaplan‐Meier method and compared using the log‐rank test. All calculations were done with StatMate V (ATMS Co., Ltd., Tokyo, Japan), or SPSS version 16.0 (SPSS Inc., Chicago, IL, USA). All results are expressed as medians (range). *P *<* *0.05 was considered statistically significant.

## 3 RESULTS

### 3.1 Patient characteristics

Among 247 patients intended to be recruited for HOPS phase II studies from October 2013 to April 2016, 88 and 93 patients were considered to have R‐PC and BR‐PC, respectively. The remaining 66 patients, all of whom were confirmed to have UR‐PC initially, were enrolled in this cohort (Table 1). They consisted of 34 men and 32 women with a median age of 67 years (range, 45‐83). The primary tumor was mainly located at the head of the pancreas in 41 patients and the body or tail of the pancreas in 25 patients. The median tumor diameter was 30 mm (range, 7‐75). According to the General Rules for the Study of Pancreatic Cancer (July 2016, seventh edition),^16^ local tumor extent was classified as cT ≤3 and cT4 in 52 and 14 patients, respectively. Nodal metastasis was clinically diagnosed in 13 of 66 patients, including in three patients with metastasis to extra‐regional (para‐aortic) lymph nodes. Metastasis was suspected in 24 patients with UR‐M disease in the liver (n = 13), peritoneum (n = 10), lung (n = 1), and bone (n = 1); one patient was suspected of having both liver and peritoneal metastasis. All 10 patients with suspected peritoneal metastasis had very minor ascites detected on MDCT with an intra‐abdominal area with the density of water that was <1 cm in width. However, peritoneal metastasis was not confirmed by cytology, so these patients were thereafter considered to have ascites. Pathological confirmation of PC was established in 62 (93.9%) of 66 patients. One patient with suspected para‐aortic lymph node metastasis was considered to have UR‐LA disease due to lack of pathological confirmation. Plexus invasion on imaging was noted in 38 (90.5%) of 42 patients with UR‐LA disease and 14 (58.3%) of 24 patients with UR‐M disease (*P *=* *0.0041).

**Table 1:** Background characteristics of patients by reason for unresectability

Factor		UR (n = 66)	LA (n = 42)	M (n = 24)	*P* value
Gender	Male/female	34/32	20/22	14/10	0.4021
Age	Years, median (range)	67 (45‐83)	67 (45‐83)	67 (46‐83)	0.9415
Main tumor location	Head/body‐tail	41/25	28/14	13/11	0.3139
Tumor diameter	mm, median (range)	30 (7‐75)	30 (15‐51)	31 (7‐75)	0.9415
Clinical TNM stage^a^					
cT	≤3/4	52/14	32/10	20/4	0.7115
CH‐DU‐S‐RP	0/1	4/62	1/41	3/21	0.0974
PV	0/1	22/44	11/31	11/13	0.1034
PL	0/1	14/52	4/38	10/14	0.0041
A	0/1	40/26	24/18	16/8	0.4462
cN	0/1/M1(LYM)	53/10/3	35/6/1	18/4/2	0.5547
cM	HEP/PER/PUL/OSS	13/10/1/1		13^b^/10^b^/1/1	
Histological confirmation	Adenocarcinoma/no	62/4	41/1	21/3	0.0974
Biliary drainage	Yes/no	35/31	21/21	14/10	0.5141

Abbreviation: A, arterial system invasion; CH, bile duct invasion; DU, duodenal invasion; HEP, hepatic metastasis; LA, locally advanced; LYM, lymph node metastasis; M, metastatic; OSS, osseous metastasis; PER, peritoneal metastasis; PL, extrapancreatic nerve plexus invasion; PUL, pulmonary metastasis; PV, portal venous system invasion; RP, retropancreatic tissue invasion; S, invasion of the serosal side of the anterior pancreatic tissue; UR, unresectable.

a Japan Pancreas Society, General rules for the study of pancreatic cancer, seventh edition.

b HEP + PER (n = 1).

### 3.2 Treatments

**Figure 1:**
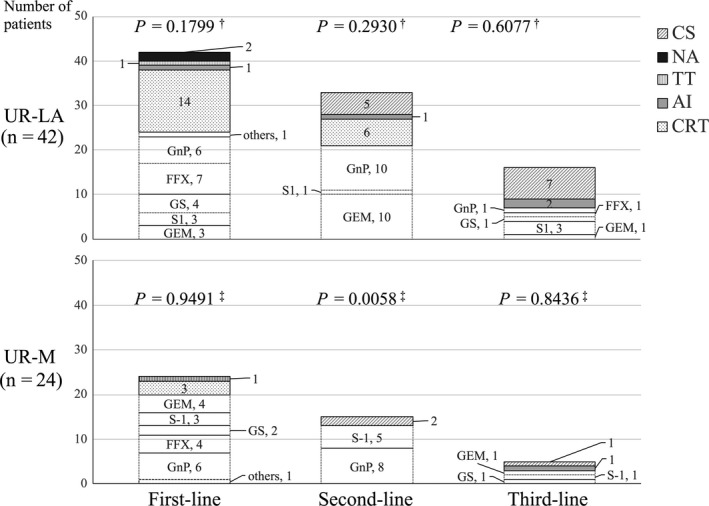
Treatment line and regimen by reason for unresectability. †*P* value for statistical trend in the distribution of treatment modalities (in the upper panel) and ‡ of chemotherapeutic regimens (in the lower panel) for each group. Data are presented as number of patients, %. AI, arterial infusion chemotherapy; CRT, chemoradiotherapy; CS, conversion surgery; CT, chemotherapy; FFX, FOLFIRINOX; Gem, gemcitabine; GnP, gemcitabine plus nab‐paclitaxel; GS, gemcitabine plus S‐1; NA, data not available; PTX, paclitaxel. TT, thermal therapy

Induction therapy was introduced with CT and CRT in 44 and 17 patients, respectively (Figure 1). CRT consisted of a total dose of 50.4 Gy divided into 28 fractions plus S‐1 (80 mg/m^2^, twice daily on radiation days). The proportion of patients who received first‐line or second‐line treatment in the UR‐LA and UR‐M groups was similar (*P *=* *0.1799). The initial CT regimen consisted of FOLFIRINOX and gemcitabine plus nab‐paclitaxel in 11 and 12 patients, respectively (Figure 1). First‐line modified FOLFIRINOX was used in 29% (seven of 24) of patients with UR‐LA disease and 20% (4 of 20) of patients with UR‐M disease. Gemcitabine plus nab‐paclitaxel was introduced in 27% (12 of 44) of patients with UR‐PC as first‐line treatment and used as second‐line treatment in 53% (18 of 34) of patients (UR‐LA, 48%; UR‐M, 62%). Second‐line gemcitabine monotherapy was significantly more commonly used in patients with UR‐LA versus UR‐M disease (*P *=* *0.00295). Third‐line modified FOLFIRINOX or gemcitabine plus nab‐paclitaxel therapy was adopted at a lower rate (20%) compared to the overall adoption rate for first‐ and second‐line therapies (52.6%; *P *=* *0.052), suggesting treatment toxicity (Figure 1).

### 3.3 Conversion to radical surgery and surgical outcomes

Surgery was not recommended for 51 of 66 patients with UR‐PC. Median progression‐free survival in these patients was estimated to be 5.7 months (95% confident interval [CI], 4.7‐6.8). Twenty‐one patients (12 with UR‐LA and nine with UR‐M disease) had disease control for more than 6 months. Fifteen patients developed progressive disease and six patients continued to have PR or SD for 11.8‐44.8 months until the latest follow‐up. The reasons why surgery was not recommended for these six patients included persistent liver metastasis in two patients, persistent ascites in one patient, and plexus involvement in three patients that extended into the superior mesenteric (n = 2) and common hepatic arteries (n = 1). Radical surgery was performed in the remaining 15 patients (12 patients with UR‐LA disease and three with UR‐M disease; Figure 1) but completed in 12 (10 patients with UR‐LA disease and two patients with UR‐M disease; Table 2). Three patients with metastasis at the time of surgery underwent palliative procedures that included probe laparotomy, choledochojejunostomy, and laparoscopic gastrojejunostomy. The median preceding treatment interval was 10.3 months (range, 2‐32) in patients with completed radical surgery (UR‐LA, 11.0 months; UR‐M, 17.8 months; *P = *0.0885). Two patients with UR‐M disease diagnosed with liver metastasis on EOB‐MRI underwent primary tumor resection because those metastases disappeared after first‐line and second‐line treatment.

**Table 2:** Summary of patients who underwent CS

Factor		CS (n = 12)
Gender	Male/female	7/5
Age	Median (range)	67 (45‐73)
Reason for unresectability	LA/M	10/2
Treatment response^a^	PR/SD	8/4
Interval between initial treatment and CS	Months, median (range)	10.3 (2‐32)
UR‐LA	Months, median (range)	11.0 (2‐32)
UR‐M	Months, median (range)	17.8 (9‐26)
Surgical procedure		
Pancreaticoduodenectomy		11
Combined vascular resection	PV‐SMV	5
	PV‐SMV + SpA	1
	PV‐SMV + HA	1
Distal pancreatectomy		1
Surgical morbidity and mortality^b^	II/IIIa/V	4/1/0
Length of postoperative hospital stay	Day, median (range)	29 (13‐44)
Pathological findings^c^		
pT	0/1/3	3/1/8
pN	0/1	10/2
pStage	‐/I/IIA/IIB	3/1/5/3
Residual tumor	R 0/1	11(91.6%)/1
Evans grade	I/IIa/IIb/III/IV	1/4/3/1/3
Adjuvant treatment	S‐1	7/12 (58.3%)

Abbreviations: CS, conversion surgery; HA, hepatic artery; PR, partial response; PV, portal vein; SD, stable disease; SMV, superior mesenteric vein; SpA, splenic artery.

a Response evaluation criteria in solid tumors.

b Clavien‐Dindo classification.

c Japan Pancreas Society, General Rules for the Study of Pancreatic Cancer, seventh edition.

The conversion rate to surgery overall and among patients with UR‐LA or UR‐M disease was 18.2%, 23.8%, and 8.3%, respectively (UR‐LA vs UR‐M, *P *=* *0.193). Operative procedures included pancreaticoduodenectomy in 11 patients and distal pancreatectomy in one patient. Concomitant vascular resection was performed: portal vein‐superior mesenteric vein (PV‐SMV) resection (n = 5), PV‐SMV plus splenic artery resection (n = 1), and PV‐SMV plus hepatic artery resection/reconstruction (n = 1). Surgical morbidity (Clavien‐Dindo grade ≥II) and mortality (Clavien‐Dindo grade V) occurred in 41.2% and 0% of patients, respectively. Postoperative length of hospital stay for patients completing radical surgery was 29 days (range, 13‐44). Pathological examination of resected specimens revealed that 33.3% (4 of 12) of patients had pT stage ≤1 and 83.3% (10 of 12) had pN stage 0 disease. The final TNM stage was I in one patient, IIA in five patients, and IIB in three patients. Eleven patients (91.6%) achieved pathological R0 resection. Four patients (33.3%) had an Evans grade ≥III response, including three patients with pathological CR (Evans grade IV). Data on individual patients who underwent CS are listed in Table S1.

### 3.4 Predictors for conversion

Among background patient characteristics, various clinicopathologic parameters, and treatment history, none were identified as significant predictors of conversion (Table S2). Interestingly, patients with CS had neither nodal nor peritoneal metastasis on initial imaging (Table S1).

### 3.5 Oncological outcome

**Figure 2:**
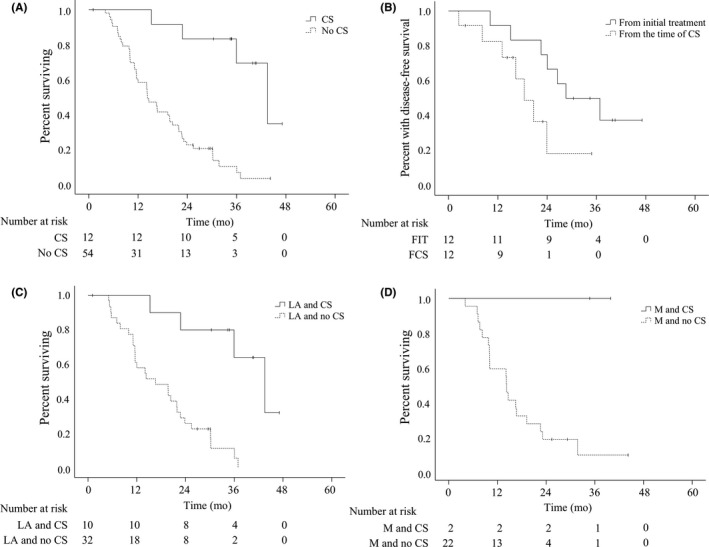
Kaplan‐Meier plots for survival by CS status and reason for initial unresectability. A. Overall survival by CS. Patients who completed CS had significantly longer MS than those who did not (44.1 vs 14.5 months, *P *<* *0.0001). B. Disease‐free survival of patients with CS from time of initial treatment and time of CS, respectively. Median duration from CS to initial recurrence was estimated to be 18.7 months. Median duration from initial treatment to initial recurrence was estimated to be 29.0 months. C. Overall survival of patients with CS stratified by initial UR‐LA status. MS of patients with initial UR‐LA‐PC who completed CS was 41.4 months, significantly longer than in patients who did not undergo surgery (16.9 months, *P *=* *0.00002). D. Overall survival of patients with CS stratified by initial UR‐M status. MS of patients with initial UR‐M‐PC who completed CS was not reached, compared with 14.4 months in those without surgery. ( | ) denotes a censored case. CS, conversion surgery; FCS, from the time of CS; FIT, from initial treatment; LA, locally advanced; M, metastatic; MS, median survival; PC, pancreatic cancer; UR, unresectable

As of the latest follow‐up, 51 (77.3%) of 66 patients had died, including 32 patients (76.2%) with UR‐LA disease and 19 patients (79.2%) with UR‐M disease. Median survival (MS) overall and among patients with UR‐LA or UR‐M disease was estimated to be 19.5, 22.2, and 14.5 months, respectively (UR‐LA vs UR‐M, *P *=* *0.366). Among patients who underwent CS, four (33.3%) of 12 patients died. Among 54 patients who did not undergo CS, 47 (87.0%) deaths occurred, suggesting that patients who completed CS had significantly longer OS than patients who did not undergo CS (44.1 vs 14.5 months, *P *<* *0.0001; Figure 2A). Seven (58.3%) out of 12 patients with CS received adjuvant CT with S‐1, but postoperative recurrence was noted in seven patients (58.3%). Median duration from CS to initial recurrence was estimated to be 18.7 months (range, 2.5‐24.3). Median duration from initial treatment to initial recurrence was estimated to be 29.0 months (range, 10.3‐37.3; Figure 2B, Table 3). The initial sites of recurrence included the liver (n = 2), peritoneum (n = 2), remnant pancreas (n = 2), lung (n = 1), and lymph node (n = 1; Table 3). Metastasectomy was performed in patients who initially recurred in the remnant pancreas (remnant distal pancreatectomy, n = 2) and lung (partial pneumonectomy, n = 1). Those three patients were all alive without additional recurrences at the latest follow‐up for 11.7, 13.1, and 4.7 months after metastasectomy, respectively. The remaining five patients did not have recurrence and were alive with a median follow‐up of 15.9 months (range, 4.1‐35.3) after surgery. Subgroups analysis of patients with CS stratified by initial UR‐LA versus UR‐M status revealed that MS in patients who underwent CS was 41.4 months and not reached, respectively, while MS of patients who did not undergo CS was 16.9 and 14.4 months, respectively (Figure 2C,D).

**Table 3:** Details about the seven patients with postoperative recurrence

Site of initial recurrence (n = 7)	n (%)	Period from CS to relapse, months, median (range)
Liver	2 (28.6)^a^	16.3 (8.3‐24.3)
Peritoneum	2 (28.6)^a^	5.4 (2.5‐8.3)
Remnant pancreas	2 (28.6)	14.9 (13.2‐16.6)
Lung	1 (14.3)	18.7
Lymph node	1 (14.3)	21
Overall		18.7 (2.5‐24.3)
Period from initial treatment to relapse, months, median (range)		29 (10.3‐37.3)

Abbreviation: CS, conversion surgery.

a Initial recurrence was noted in both the liver and peritoneum simultaneously.

**Figure 3:**
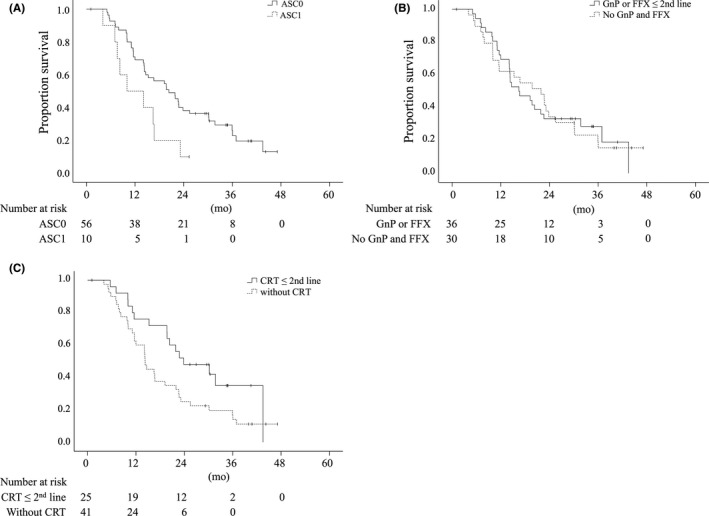
Kaplan‐Meier plots for overall survival stratified by subgroup. A. Survival of patients with and without ascites on diagnostic imaging (ASC). Patients with ascites (ASC1) had significantly worse MS than patients without ascites (ASC0; MS, 10.2 vs 20.6 months, *P *=* *0.0257). B. Survival of patients with and without chemotherapeutic regimens such as FOLFIRINOX and gemcitabine plus nab‐paclitaxel as second‐line treatment were comparable (MS, 16.7 vs 22.2 months; *P *=* *0.9482). C. Patients with CRT as second‐line treatment had significantly better MS than those without (MS, 24.2 vs 14.5 months; *P *=* *0.0455). ( | ) denotes a censored case. ASC, ascites; CRT, chemoradiotherapy; MS, median survival

Next, prognostic factors related to OS were analyzed using Cox proportional hazards modeling. CS (hazard ratio [HR], 0.078; 95% CI, 0.017‐0.348; *P *=* *0.001) was a significant predictor of longer OS, and ascites on diagnostic imaging was a marginal predictor of shorter OS (HR, 2.192; 95% CI, 0.967‐4.969; *P *=* *0.060; Table 4). Subgroup analyses showed that survival of patients with ascites on diagnostic imaging was significantly worse compared with patients without this finding (MS, 10.2 vs 20.6 months; log‐rank *P *=* *0.026; Figure 3A). Modern chemotherapeutic regimens such as FOLFIRINOX or gemcitabine plus nab‐paclitaxel as second‐line treatment had comparable survival (MS, 16.7 vs 22.2 months; log‐rank *P *=* *0.9482; Figure 3B). However, patients with CRT as second‐line treatment had significantly better MS than those without (24.2 vs 14.5 months; log‐rank *P *=* *0.046; Figure 3C). It is obvious that there was bias in treatment selection because CRT as second‐line treatment was used in eight (75%) of 12 patients who underwent CS, compared with 15 (27.8%) of 54 cases who did not undergo CS (*P *=* *0.0265). In terms of treatment response, no association between histopathological tumor response (Evans ≥III vs <III) and prognosis of patients with UR‐PC was observed (log‐ rank *P *=* *0.112, data not shown).

**Table 4:** Univariate and multivariate analyses for factors predictive of overall survival

Factors		Univariate			Multivariate		
		Exp (ß)	95% CI	*P* value	Exp (ß)	95% CI	*P* value
Gender	Male vs female	1.008	0.561‐1.812	0.978			
Age, years	<67 vs ≥67	0.900	0.492‐1.648	0.733			
Histological confirmation	Adenocarcinoma	1.227	0.426‐3.536	0.705			
Main tumor location	Head vs body‐tail	0.601	0.322‐1.122	0.110	0.510	0.227‐1.146	0.103
Tumor diameter	<30 vs ≥30 mm	1.225	0.670‐2.237	0.510			
Clinical TNM stage^a^							
cT	≤3 vs 4	0.740	0.342‐1.600	0.443			
PV	0 vs 1	0.671	0.365‐1.234	0.200			
PL	0 vs 1	0.659	0.338‐1.288	0.223			
A	0 vs 1	0.749	0.402‐1.396	0.363			
cN	0 vs 1	1.956	0.996‐3.840	0.051	1.055	0.506‐2.199	0.887
cM	0 vs 1	1.757	0.949‐3.252	0.073			
ASC	0 vs 1	2.498	1.181‐5.282	0.017	2.192	0.967‐4.969	0.060
HEP	0 vs 1	0.948	0.438‐2.050	0.892			
Biliary drainage	Yes/no	1.666	0.917‐3.027	0.094	1.473	0.682‐3.182	0.324
Reason for unresectability	LA vs M	1.667	0.899‐3.093	0.105			
Conversion surgery	Yes/no	0.087	0.021‐0.364	0.001	0.078	0.017‐0.348	0.001
Treatment ≤ second‐line	mFFX or GnP	1.106	0.601‐2.036	0.746			
	CRT	0.512	0.263‐0.999	0.050	0.815	0.407‐1.633	0.564

Abbreviation: A, arterial system invasion; ASC, ascites on diagnostic imaging; CRT, chemoradiotherapy; GnP, gemcitabine plus nab‐paclitaxel; HEP, hepatic metastasis; LA, locally advanced; M, metastatic; mFFX, modified FOLFIRINOX; PL, extrapancreatic nerve plexus invasion; PV, portal venous system invasion.

a Japan Pancreas Society, General Rules for the Study of Pancreatic Cancer, seventh edition.

## 4 DISCUSSION

This multicenter, retrospective HOPS cohort study demonstrated that OS of patients who were clinically diagnosed with UR‐PC and completed CS after successful multidisciplinary induction treatment is significantly longer than OS in patients who did not, suggesting that a certain population of patients with UR‐PC actually benefit from CS. This study stands out in that it focuses on patients initially diagnosed with R‐PC or BR‐PC based on MDCT, resulting in a group with no apparent distant metastasis but suspicious or very small amounts of M disease identified by central reviewers. Those patients are usually less likely to become candidates for radical surgery; however, CS was performed at five leading HOPS institutions with favorable perioperative results. MS in those patients, starting from initial treatment, was estimated to be 44.1 months (95% CI, 32.9‐55.3). The clinical efficacy of CS is strongly suggested by these survival results in a multicenter setting.

Several studies on CS among patients with UR‐PC have been reported so far.^6^, ^8^, ^9^, ^11^, ^17^, ^18^, ^19^, ^20^, ^21^ In the most recent meta‐analysis based on reports since 2009, the rate of conversion from UR‐LA disease to surgery was 26% and OS was 18.7 months.^10^ Other reports of conversion from initial UR‐PC to radical surgery after favorable response to induction treatment reported a wide range of conversion rates, depending on whether patients with M disease were included.^9^, ^11^, ^17^, ^20^, ^21^ Nitsche et al^17^ reported that CS was possible in four (28.6%) of 14 patients with UR‐PC who received FOLFIRINOX as first‐line treatment. On the other hand, Hackert et al^21^ reported that CS could be performed in 292 (50.8%) of 575 patients with UR‐PC who received neoadjuvant FOLFIRINOX. In this multicenter retrospective study, the current cohort mostly comprised of patients with LA‐PC without obvious distant metastasis on diagnostic imaging. The rate for conversion was 18.2% overall, 23.8% in patients with UR‐LA, and 8.3% in UR‐M disease, suggesting that conversion rates for LA‐PC and M‐PC are approximately 20%‐30% and <10%, respectively, in real‐world clinical practice.

To date, there have been no definitive reports concerning predictive factors for conversion to radical surgery in patients with LA‐PC and M‐PC following induction treatment.^9^, ^20^ Various prognostic factors for OS, on the other hand, have been identified in prospective and retrospective studies.^22^, ^23^ Abendroth et al^22^ demonstrated that the number of treatment lines is an independent predictor of long‐term outcomes in patients with M and LA‐PC before the era of modern combination therapy protocols. Choi et al^23^ analyzed survival in patients with LA‐PC treated with CRT; they found that the administration of high‐dose radiation (≥61 Gy), maximum standard uptake value on initial PET‐CT (<3.5) with carbohydrate antigen (CA) 19‐9 ≤400 U/mL, and surgical resection after CRT were significantly related to prolonged OS based on multivariate analysis. There is consensus that completion of radical surgery after induction treatment is one of the leading prognostic factors for OS in patients with UR‐PC, irrespective of LA or M status.^6^, ^9^, ^20^ However, it still remains unclear whether the prolongation of OS resulted from completing surgery or long‐term patient selection during induction CT. Interestingly, MS of patients with minor ascites on MDCT suggestive of latent peritoneal metastasis was shorter in the current multicenter cohort study, suggesting that ascites should be considered before initiating induction treatment for potential UR‐LA‐PC. This patient population might benefit from staging laparoscopy to discriminate between false‐positive imaging findings and peritoneal metastasis.

In the current study cohort, postoperative recurrence occurred in more than half of patients with a median duration from CS to initial recurrence of 18.7 months, although the R0 resection rate was 91.6% with pathological Evans grades III‐IV disease in four patients. Some patients had earlier recurrence after CS. Peritoneal metastasis occurred the earliest, with a median time to relapse after CS of 5.4 months. For patients with early postoperative recurrence within 18 months, corresponding to the median OS with the current nonsurgical cohort, highly invasive CS cannot be considered an effective treatment. Further studies including genomic or molecular approaches are necessary, as well as liquid biopsy to detect latent distant metastasis.^24^, ^25^ These efforts might facilitate identifying patients at higher risk for early disease relapse after surgical resection and lead to treatment decisions for more intensive therapies to eliminate subclinical, residual, and latent disease in patients with initial UR‐PC after induction treatment.

Whether to convert from initial chemo(radio)therapy to surgery solely on the basis of radiological examination remains controversial. Several authors have documented a significant association between preoperative CA19‐9 values and sub‐radiographic, UR‐PC with systemic metastases.^8^, ^26^, ^27^ The CA19‐9 response to neoadjuvant therapy has been reported to be another potential marker for R0 resection, histopathologic response, and survival,^26^ suggesting that CA19‐9 levels should be taken into account when evaluating the efficacy of CT. ^18^F‐fluorodeoxyglucose PET findings could also be a potential indication for CS in patients with primary UR‐LA‐PC, and may help in selecting patients who qualify for complete surgical resection with a promising prognosis.^27^ Future studies should assess how to select patients for CS and whether CS after initial induction treatment improves OS.

This current study has some limitations. It was a retrospective study with a small number of patients who underwent CS. There was likely some bias in the selection of CT regimens due to the concomitant HOPS‐BR01 study involving second‐line gemcitabine monotherapy, suggesting possible inconsistencies in decision‐making with regards to subsequent treatment lines or indications for surgery. Decision‐making at the discretion of the attending physician for each patient was the major reason why an analysis of the best timing for surgical conversion could not be performed. The predictors of conversion were not determined, perhaps because the series of treatments was not systematically determined. In our hospital, decision‐making has been largely standardized based on multimodal treatment conferences since 2012.^9^ Another limitation of this study is that initial PC status was evaluated with only MDCT and that neither tumor markers such as CA19‐9 nor staging laparoscopy were performed. It was not possible to evaluate changes in tumor markers objectively in relation to treatment effect or prognosis since the CA19‐9 measurement protocol was not standardized between participating hospitals. Comprehensive treatment evaluation with tumor markers plus diagnostic imaging might more accurately predict the timing to surgical conversion or prognosis. Staging laparoscopy allows for the diagnosis of minute distant organ metastasis^28^ but was not performed in this cohort, suggesting overestimation of suspected peritoneal metastasis or undetected latent distant metastasis in UR‐LA‐PC. Recently, international consensus on the definition and criteria for BR‐PC was defined according to three distinct dimensions: anatomical, biological, and conditional.^29^ This definition acknowledges that resectability is not just about the anatomic relationship between the tumor and vessels, but that biological and conditional dimensions including the status of the tumor marker CA19‐9 (> or ≤500 units/mL) and performance status are also important. In order to improve OS, future studies should assess whether to consider CS after initial induction treatment for patients with initial UR‐PC and its timing. In Japan, the PREP‐04 trial (UMIN 000017793), a multi‐institutional prospective observational study to investigate the effects of CS in patients with initial UR‐PC, is already ongoing.

## 5 CONCLUSION

Conversion surgery following a favorable response to sequential treatment may be a good option to prolong survival in patients with UR‐PC. Precise imaging diagnosis based on MDCT followed by sequential multimodal anticancer treatment is essential.

## DISCLOSURE

Conflict of interests: authors declare no conflict of interests for this article.

Author Contributions: Kimura and Imamura had full access to all the data in the study and take responsibility for the integrity of the data and the accuracy of the data analysis. Study concept and design: Kimura, Imamura, Yamaguchi, Motoya, and Yoshida. Acquisition or interpretation of data: Kimura, Imamura, Yamaguchi, Motoya, Yoshida, Nagayama, Yamakita, Goto, and Takahashi. All MDCT interpretations were performed by Sakuhara and were verified by Nakamura and Kuwatani. Drafting of the manuscript: Kimura, Yamaguchi, and Takemasa. Critical revision of the manuscript for important intellectual content: Maguchi, Hirano, and Takemasa. Administrative, technical, or material support: Maguchi. Study supervision: Hirano.
